# Cultivation mode of new accounting talents in the context of financial and taxation digitalization

**DOI:** 10.1371/journal.pone.0276005

**Published:** 2022-11-09

**Authors:** Shuangshuang Wu

**Affiliations:** School of Business and Management, Neusoft Institute Guangdong, Foshan, 528225, China; Asia University, TAIWAN

## Abstract

With the development of digital economy, the degree of enterprise digitalization is regarded as the core competitiveness. Therefore, cultivating financial and digital accounting talents has become the mainstream trend. This paper determines the weight of each factor through the entropy right method, and sorts the importance of the evaluation index of the new accounting talent training. On this basis, the factors affecting the training of new accounting talents are analyzed and evaluated. In order to adapt to the development of digital economy society, cultivate compound accounting talents with cross-border knowledge for the society.

## 1. Introduction

As the social economy accelerates into the digital age, digital technology has not only brought about the revolution of production technology and the change of business model. And the tentacles have been extended to all walks of life. In recent years, new technologies have been gradually combined with the economy and developed very rapidly. Such as artificial intelligence, blockchain, cloud computing and big data. The continuous emergence of these innovative technologies has accelerated the gradual and deep integration of the real economy and digital technology [1]. This fusion enables numbers to use knowledge and information as the factors of production, and information technology as the carrier. Finally, the digital economy that uses communication technology to improve efficiency is booming. At the same time, the new generation of information technology and the three major industries are integrated. We will form a new economic form with new technologies, new industries, new forms of business and new models as the core [2]. According to the survey, the data economy has produced a subversive effect on the financial and accounting function in many aspects [3]. According to the survey, 58 percent of the respondents said they need to deepen their understanding of digital, intelligent technology and complex data analysis technology. Delivery and advanced analysis technologies will be the most needed key capabilities for future financial functions. Fifty-seven percent of the treasurers believe that risk management will be a key capability in the future and will increasingly be held accountable for the ethical nature of the relevant decisions underpinning the financial goals [4].

The so-called digital economy is the direct or indirect use of data to guide and realize the optimal allocation and regeneration of accounting resources. The economic form of realizing high-quality economic development. Rapid and large-scale support and realize the overall transformation and upgrading of enterprises of different sizes. Help enterprises to improve the management level, reduce cost and increase efficiency [5]. In order to have a new core competitiveness, enterprises must carry out digital transformation. Therefore, in the transformation process, the financial institutions and their personnel are crucial to the digital operations [6].

Cultivating new accounting talents needs to conform to the development of science and technology and economy. In order to improve the comprehensive quality and ability of accounting practitioners, improve the service quality of the accounting industry. Compared to the traditional culture model of manual bookkeeping. In the era of big data, digital fiscal and tax talents need to pay more attention to the comprehensive training of their own knowledge, ability and quality. Zhou Hong has constructed a basic and perfect ability framework for different levels and positioning of accounting talents according to different ability elements,——knowledge, skills and values. Define different levels by primary, intermediate, and advanced. To define different positioning by business operation, accounting supervisor and operation management [7]. The capability framework is basically formed. The disadvantage is that the evaluation of talents is too subjective, and it cannot be effectively promoted without hard and objective data. It is necessary to strengthen the training of new accounting talents. Therefore, it is particularly important to evaluate the accounting students majoring in colleges and universities. Now although many evaluations have some quantitative methods, but cannot make a scientific and reasonable analysis of accounting talents. The practice and measures to improve the training of new accounting talents become mere formality and the evaluation is not specific enough [8–12].

Therefore, this paper will discuss the new mode of accounting talent training under the background of finance and tax digitization. Taking the accounting experimental class of a university as the research object and using the method of questionnaire survey. Qualitative analysis and quantitative calculation are performed by introducing the entropy weight method and the fuzzy comprehensive evaluation method. Analyze and evaluate the training mode of new accounting talents. And discusses the evaluation index system and its influencing factors. To promote the cultivation of finance and tax digital talents. Provide scientific basis for the training mode of new accounting talents. And actively cultivate digital talents to adapt to the current social and economic development.

## 2. Study design and data processing

The study was approved by the ethics committee of Neusoft Institute Guangdong.

### 2.1 Data collection

This paper takes a university accounting experimental class as a research object. On the premise of clarifying the training objectives, according to the knowledge, ability and quality of the school training program level concept indicators. With literature studies [12] and the Delphi method, the second-level evaluation indicators are determined. The ting of evaluation indicators through questionnaire survey and hierarchical analysis. To form a new accounting talent training system consisting of 4 first-level indicators and 9 second-level indicators [13]. The survey was conducted by questionnaire survey. Fifty questionnaires were issued, and 48 questionnaires were recovered, with a recovery rate of 96%. In order to understand the study of the subjects’ understanding of the importance of various training evaluation indicators.

Based on the purpose of the study and the availability of data, the questionnaire was designed using the Likert scale 1–5 level. Collect the evaluation grade scores of the respondents on the new accounting talent training program. The scoring pattern of the questionnaire design was scored according to the importance of each factor index. The score was used as the evaluation data. Where, 5 is important, 4 important, 3 general, 2 unimportant, and 1 very unimportant.

### 2.2 Research methods

This study uses the entropy weight method and the fuzzy comprehensive evaluation method. Entropy was first a thermodynamic concept developed by the German physicist R.Clsusius. The entropy weight method mainly uses the uncertainty represented by entropy values in consulting theory. To calculate the evaluation attributes can transfer the decision consultation ability, and calculate the relative weight between the attributes. The entropy weight method is objectively delegated. The weight serves as the weight vector of the fuzzy comprehensive evaluation method. Since the same thing has multiple attributes and is influenced by multiple uncertainties. Therefore, in the process of evaluation, multiple relevant factors must be comprehensively considered and must be fully evaluated [14]. That is, the membership level of the evaluated thing is determined from multiple dimensions and indicators. And conduct a comprehensive judgment. Through the mathematical model, the evaluation index of the new accounting talent training is measured.

In the traditional fuzzy comprehensive evaluation. The weight of indicators is not produced in the process of judgment, but subjectively determined by experts according to their own experience and actual judgment. The selected experts also have different weights. The main disadvantage of this weight method is subjective arbitrariness. It largely affects the differentiation degree of the evaluation results [15]. Therefore, the entropy weight method and the fuzzy comprehensive evaluation method are combined. To improve the credibility of the evaluation results.

#### 2.2.1 Entropy weight method and weight determination

Assuming m schemes to be evaluated, n evaluation indicators. Form the original index data matrix *X* = (*x*_*ij*_)_*m*×*n*_ (0≤i≤m,0≤j≤n),The entropy weight evaluation model is as follows [16]:

1. The sample *u*_*ij*_ means belonging to the k categories:
$$\mu_{ij}=x_{ij}/\sum_{j=1}^{n}x_{ij}$$
$$\mu =(\mu_{i1},\mu_{i2},\cdots ,\mu_{ijk})$$ (1)
  Satisfied 0≤*μ*_*ijk*_≤1, That is {*μ*_*ijk*_} has some probabilistic property.
2. Calculate the entropy of each indicator:
$$H=-\frac{1}{\log k}\sum_{k=1}^{k}\mu_{ijk}∙\log\mu_{ijk}$$ (2)
3. Calculate the information utility value for each indicator:
$$v_{ij}=1+\frac{1}{\log k}\sum_{k=1}^{k}\mu_{ijk}∙\log\mu_{ijk}$$ (3)
4. Calculate the weight of each index, namely the entropy weight:
$$W_{ij}=v_{ij}/\sum_{j=1}^{n}v_{ij}$$ (4)

#### 2.2.2 Fuzzy comprehensive evaluation method

Fuzzy comprehensive evaluation method is a comprehensive evaluation method based on fuzzy mathematics, transforming qualitative evaluation into quantitative evaluation according to the principle of membership. Using fuzzy mathematics to make the overall evaluation of the same object affected by multiple factors can better solve the difficult to quantify problems, especially for non-deterministic problems [17,18].

1. Establish the factor set of the evaluation object U = {*U*_1_, *U*_2_,…*U*_*m*_}:
  Factor set is a common set of various factors that affect the evaluation object, usually expressed by U. The element U_i_ represents the I th factor that affects the evaluation object. These factors usually have varying degrees of ambiguity.
2. Establish an evaluation set of comprehensive evaluation V = {*V*_1_, *V*_2_,…*V*_*m*_}:
  The evaluation set is a set of various results that the evaluator may make to the evaluation object, usually expressed by V, where the element V_j_ represents the j th species evaluation result.
3. To establish a fuzzy relationship matrix:
$$R=\left[\begin{array}{c} R_{1}\\ R_{2}\\ R_{3}\\ R_{4} \end{array}\right]=\left[\begin{array}{cccc} R_{11} & R_{12} & \cdots & R_{1k}\\ R_{21} & R_{22} & \cdots & R_{2k}\\ \vdots & \vdots & \vdots & \vdots \\ R_{m1} & R_{m2} & \cdots & R_{mk} \end{array}\right]$$ (5)
  Among R_*ij*_ (i = 1,2,…,m; j = 1,2,…,k) for the membership of the i th evaluation index to the j th evaluation level, it reflects the fuzzy relationship between the evaluation index and the evaluation grade, which is expressed by the membership degree; The m indicates m factor levels; the k indicates k evaluation levels.
4. Establish the weight set and determine the factor weight vector:
5. The importance of each factor varies, giving corresponding weights to each factor variable to obtain the set of factor weights, which is expressed by W: W = {*w*_1_, *w*_2_,…*w*_*n*_},and $\sum_{1}^{n}w_{i}=1$, w_*i*_≥0, i = 1,2,…,n. Fuzzy comprehensive evaluation
  First-level fuzzy comprehensive evaluation: After determining the factor weight vector W and the fuzzy relationship matrix R, the membership vector Y of the factor set is obtained according to the fuzzy transformation principle and through the compound operation:
$$Y_{1}=W_{1}∙R_{1}=(w_{1},w_{2},\cdots w_{n})∙\left[\begin{array}{cccc} R_{11} & R_{12} & \cdots & R_{1k}\\ R_{21} & R_{22} & \cdots & R_{2k}\\ \vdots & \vdots & \vdots & \vdots \\ R_{m1} & R_{m2} & \cdots & R_{mk} \end{array}\right]$$ (6)
  Second-level fuzzy comprehensive evaluation: Second-level fuzzy comprehensive evaluation based on first-level fuzzy comprehensive evaluation: B = W•Y = (w_1_, w_2_,⋯w_n_)∙(Y_1_, Y_2_,⋯Y_n_), Comprehensive evaluation results are obtained according to the principle of maximum membership.
6. Determine the comprehensive evaluation score: S = B•V^*T*^

### 2.3 Data processing

#### 2.3.1 Entropy right analysis of talent training evaluation index

Entropy is a measure of uncertainty in information theory. The smaller the entropy weight indicates that the greater the degree of difference in the index, the higher the weight given by the index, and the greater the role played in the evaluation.

The raw data matrix was determined using the score results of the questionnaire *X* = (*x*_*ij*_)_*m*×*n*_ (0≤i≤m, 0≤j≤n), according to the entropy weight method Formula (1) ~ (4). Calculate the weight of each index of the new accounting talent training and evaluation and the weight of the standard layer. The corresponding results of the weights are also ranked, as shown in Table 1.

**Table 1:** Evaluation index system and index weight and ranking of new accounting talent training.

First-level index layer	Weight	Second-level index layer	weight	Evaluation of grade membership					Combination weight	Sequencing of weights
				Very important	Important	General	Unimportance	Very unimportant		
Speculative knowledge	0.238	Basic knowledge	0.259	0.250	0.396	0.208	0.104	0.042	0.062	8
		Professional theory	0.741	0.479	0.333	0.188	0.000	0.000	0.176	1
Skills qualification	0.232	Specialized skill	0.473	0.375	0.375	0.104	0.146	0.000	0.110	5
		Qualification certificate	0.527	0.271	0.458	0.208	0.063	0.000	0.122	4
Ability to apply	0.269	Professional proficiency	0.531	0.313	0.438	0.229	0.021	0.000	0.143	3
		Interpersonal competence	0.224	0.313	0.250	0.313	0.083	0.042	0.060	9
		Knowledge application ability	0.245	0.333	0.354	0.188	0.063	0.063	0.066	7
Quality level	0.261	Moral quality	0.606	0.375	0.333	0.292	0.000	0.000	0.158	2
		Professional quality	0.394	0.271	0.396	0.250	0.083	0.000	0.103	6

#### 2.3.2 Calculation of fuzzy comprehensive evaluation

1. Each second-level index is evaluated at 5 levels: "very important", "important", "general", "unimportant" and "very unimportant". That is, the evaluation level V = {V_1_, V_2_, V_3_, V_4_, V_5_} = {very important, important, general, not important, very unimportant}. To assign its value V = {95, 85, 75, 65, 55}.
  The membership of the evaluation index was obtained after normalization, and the results are shown in Table 1. After synthetic operation, the first-level fuzzy comprehensive evaluation results are obtained. Taking theoretical knowledge as an example, the weight vector of theoretical knowledge Y_1_ is:
$$Y_{1}=W_{1}∙R_{1}=(0.259,\;0.741)∙\left[\begin{array}{ccccc} 0.250 & 0.396 & 0.208 & 0.104 & 0.042\\ 0.479 & 0.333 & 0.188 & 0.000 & 0.000 \end{array}\right]=(0.420,\;0.349,\;0.193,\;0.027,\;0.011)$$
  Similarly, the weight vectors of the remaining first-level index factors can be found:
$$Y_{2}=(0.320,\;0.419,\;0.159,\;0.102,\;0.000)$$
$$Y_{3}=(0.318,\;0.375,\;0.238,\;0.045,\;0.025)$$
$$Y_{4}=(0.334,\;0.358,\;0.275,\;0.030,\;0.000)$$
2. According to the formula of the second-level fuzzy comprehensive evaluation, the weight vector of the evaluation results of the new accounting talent training is obtained:
B=(0.347,0.375,0.219,0.051,0.009).
3. According to the formula of the comprehensive evaluation score, the comprehensive evaluation score is obtained:
$$S=B\cdot V^{T}={\left(0.347,\;0.375,\;0.219,\;0.051,\;0.009)∙(95,\;85,\;75,\;65,\;55\right)}^{T}=84.99$$

## 3. Study results and outlook

**Fig 1:**
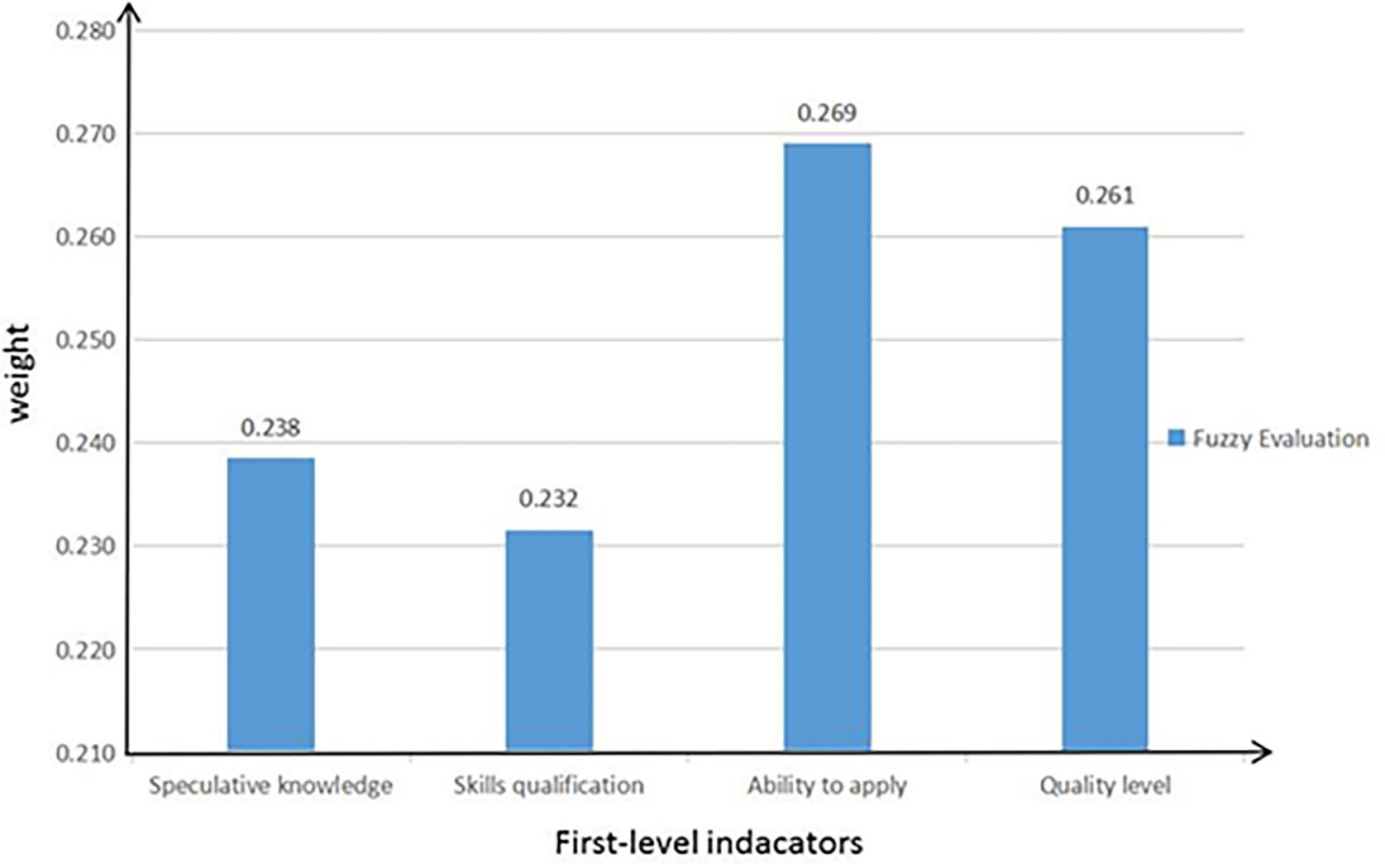
Fuzzy comprehensive evaluation of the new accounting talent training mode.

Based on the weight ratio of the four first-level indexes calculated by the entropy weight method is in order ability application 0.269, quality level 0.261, theoretical knowledge 0.238, skill qualification 0.232. As shown in Figs 1, compared with other indicators, capability applications are ranked first. It shows that the cultivation of new accounting talents should focus on the application of ability. Therefore, in practice, we should pay attention to cultivating accounting talents’ business ability, interpersonal ability and knowledge application ability. It can be seen from the weight ranking of the 9 second-level index system of the new accounting talent training. Professional theory comes first. It shows that with the development of society and the form of digital economy, the professional theoretical knowledge of new accounting talents needs to be focused on training.

By combining subjective and objective empowerment, the selection of various index factors can be comprehensively analyzed. Increase the credibility of the weight, so as to enhance the scientific nature of the evaluation. The fuzzy comprehensive evaluation shows that the membership degree of the new accounting talent training is "very important" is 0.347, "important" membership was 0.375. The "generic" membership level is 0.219. The membership of "unimportant" was 0.051. The "very unimportant" membership is 0.009. From the evaluation of the comprehensive score description. The comprehensive evaluation score of the new accounting talent training and evaluation index system based on the entropy right method and the fuzzy comprehensive evaluation method is 84.99. The evaluation results are ranked between important and general. According to the principle of maximum membership, the training mode of the new accounting talents is important. It reflects the connotation of the evaluation index of the new accounting talent training. The feasibility and practicability of the evaluation theory are verified. It shows that the system makes the evaluation results more objective and practical value.

The construction of a new digital accounting talent training mode is first conducive to changing the traditional accounting talent training concept. The traditional accounting talent training mode and theoretical teaching are difficult to be qualified for the compound requirements of accounting talents under the new economic model. Under the new economic model, the accounting will no longer learn the simple accounting theoretical knowledge as the main purpose, but the compound talents with multi-field knowledge. Therefore, the training mode of new accounting talents should subvert the tradition. Realize the effective integration and optimization of multi-field theoretical courses and teaching resources. Focus on cultivating digital accounting skills under the new economic model. With lecture and special courses, aiming at the current hot topics in enterprise management, financial accounting and other aspects. Combined with the actual social operation situation, hire well-known professors of the industry and enterprises, business managers and students to share practical experience. Expand students ’vision, and cultivate students’ ability to analyze and solve cutting-edge problems and complex problems. In the actual teaching process, various teaching methods such as teacher teaching, student discussion, case teaching and simulation practice can be used to stimulate students’ interest in learning [19]. To realize the enterprising digital accounting talent training mode in cross-border knowledge, comprehensive ability, learning attitude, values and other aspects. Digital accounting professionals not only have a solid theoretical knowledge of accounting professional knowledge and IT digital core knowledge. At the same time, through enterprise operation simulation and practical training, it also has the ability to analyze and solve fiscal and tax related problems.

Secondly, it is conducive to impart the most cutting-edge knowledge of finance and tax digitization. Fiscal and tax digitization is data-based to maximize the value of data. The cultivation of digital accounting talents should subvert the traditional accounting textbooks [20]. In the context of the digital age, in addition to the basic theoretical knowledge of accounting. A large amount of content is to teach the most cutting-edge fiscal and tax data application, electronic invoice, fiscal and tax risk control and other practical knowledge in the digital era. Train students how to collect and sort out data, screen and analyze data, issue financial and tax operation reports and manage electronic invoices; Follow the latest national fiscal and tax policies, full simulation of electronic tax bureau simulation for online tax declaration; Find out and prevent potential fiscal and tax risks through data analysis. The design of the course is the combination of finance and taxation digitization and the corresponding accounting theory knowledge. On the basis of learning theoretical knowledge, online and offline teaching and practice of finance and taxation. Accounting theory knowledge is the basis of finance and taxation digitization. The digitalization of finance and taxation has consolidated the foundation of accounting theory. They go hand together and promote each other. Through the combination of different modules, such as textbook knowledge, finance and tax platform, and social practice. Improve the ability and quality level of accounting talents. To achieve the training goal of innovative and practical composite digital accounting talents [21–24].

Finally, let companies play the biggest role. Competition to promote teaching, to promote learning by competition. Further deepen the school-enterprise cooperation system. Realize the practical teaching mode of "software simulation-simulation-off-campus practice". Explore the sustainable cooperation mechanism of colleges and enterprises. Combine the characteristics of colleges and universities, cultivate a number of stable cooperative enterprises. Provide a fixed place for student simulation and off-campus practice [25]. It can introduce the enterprise real business to realize the seamless connection of theory and practice. Appoint the enterprise high-skilled personnel to give lectures. "Double training subject" ensures that the institute has access to real business on campus. It can also let the students exercise in the actual enterprise, to achieve comprehensive skills training and information technology improvement. Schools and enterprises form an education governance community, trained in multiple ways, and staffed. Enterprises cooperate to establish a "master studio" and an "expert workstation". Colleges and universities should adapt to the development needs of "Internet + ". Using the Internet technology to improve the classroom teaching methods. We will accelerate the construction of online learning Spaces such as virtual classrooms and virtual factories [26].

The training of accounting talents and the teaching of theoretical knowledge in colleges and universities are always the main content of accounting talents training [27–29]. Even in line with the digital economy of the accounting talent training model change. It is still difficult to design a large number of practical opportunities into teaching programs. Then, the holding of various fiscal and tax events just makes up for the defects of students’ application in practice. Accounting society and a large number of corporate institutions engaged in accounting teaching. In order to promote the reform and innovation of accounting professional education in China. Actively explore the new perspectives, new paths and new methods of accounting professional development from different perspectives. In line with the current booming digital economy. In order to promote innovation to realize the deep integration of big data, blockchain and other information technology and accounting professional education. To train high-quality and professional fiscal and tax accounting talents to meet the needs of social and economic development. Every year, various accounting skills competitions, ERP sand table competitions, financial analysis and decision-making competitions are held [30]. In the process of preparing for the competition, the students set up groups, find out information after school, and comprehensively use their knowledge of accounting theory and other fields. Change from passive learning to active learning. Such a competition is the most practical simulation practice for students before entering the society. Integrate the knowledge of all subjects learned. The students’ theoretical knowledge has been systematically sublimated. The overall improvement of the students’ ability and quality. Colleges and universities have promoted teaching through competition, and students have promoted learning by competition [31].

## 4. Summary

This paper studies the training mode of new accounting talents. Evaluation results based on entropy and weight method and fuzzy comprehensive evaluation method. It is necessary to improve the ability and quality level of the new accounting talents. Although the research method has subjective arbitrariness, it will affect the differentiation degree of the evaluation results to some extent. However, in the background of financial and tax digitization, the training objectives of schools and the training programs of enterprises can also reflect its importance.

The future training of future accounting talents should follow the trend of science and technology to cultivate digital accounting talents. Change the concept of accounting talent training. Integrate digital technology into accounting theory. Always remain sensitive and learning to changes in accounting standards, and have proficiency in using digital technology.
